# Neutrophil to high-density lipoprotein cholesterol ratio is positively associated with congestive heart failure among hypertensive adults: a population-based study

**DOI:** 10.1186/s12872-026-06177-x

**Published:** 2026-07-23

**Authors:** Kang Huang, Bo Wu, Xin Hong, Shuai-shuai Zhao, Cheng-tao Peng, Hai-chang Xu, Chuan Yuan, Wen-jing Wang, Qi-cai Wu, Xue-liang Zhou

**Affiliations:** 1 Department of Cardiovascular Surgery, The First Affiliated Hospital, Jiangxi Medical College, Nanchang University, Nanchang, 330006 China; 2 Department of Breast Surgery, The First Affiliated Hospital, Jiangxi Medical College, Nanchang University, Nanchang, 330006 China

**Keywords:** Congestive heart failure, Neutrophil to high-density lipoprotein cholesterol ratio, Hypertension, Machine learning, NHANES, Cross-sectional study

## Abstract

**Background:**

Congestive heart failure (CHF) continues to pose a substantial global health issue. The neutrophil-to-high-density lipoprotein cholesterol ratio (NHR), a novel biomarker reflecting systemic inflammatory balance, has been linked to cardiovascular outcomes, yet its relationship with CHF in hypertensive individuals is unclear.

**Methods:**

A cross-sectional investigation was adopted using data obtained from the National Health and Nutrition Examination Survey (NHANES) spanning the years 1999 to 2018. Weighted multivariate logistic regression was used to evaluate the independent correlation between NHR and CHF. Weighted restricted cubic spline (RCS) curves were applied to explore possible nonlinearities in the overall population and across subgroups. Weighted stratified comparison and interaction tests were executed for robustness. Mediation analysis assessed potential intermediaries. Seven machine learning (ML) models were developed to forecast CHF probability, with performance measured by Receiver Operating Characteristic (ROC) analysis, among others.

**Results:**

A total of 19,820 individuals with hypertension were included. Elevated NHR was correlated with higher CHF prevalence. After full adjustment, NHR remained independently associated with CHF (odds ratio [OR] = 1.15, 95% confidence interval [CI]: 1.10–1.21). Individuals in the highest quartile of NHR exhibited a 69% increased prevalence (OR = 1.69, 95% CI: 1.34–2.13) of CHF when benchmarked against the lowest quartile. Weighted subgroup analyses showed consistent associations without significant interactions. Mediation analysis indicated serum albumin mediated 4.18% of the NHR-CHF association. Among ML models, Extreme Gradient Boosting (XGBoost) showed the optimal predictive efficacy [validation set Area Under the Curve (AUC) = 0.781, 95% CI: 0.758–0.803].

**Conclusions:**

Among hypertensive adults, NHR was independently associated with CHF prevalence. These findings suggest that NHR may be a relevant inflammatory marker in this population. However, the findings should be interpreted cautiously because of the cross-sectional design and the reliance on self-reported CHF.

**Supplementary Information:**

The online version contains supplementary material available at https://doi.org/10.1186/s12872-026-06177-x.

## Background

With ongoing changes in environmental factors and lifestyle, the incidence of congestive heart failure (CHF) has been steadily rising, affecting over 26 million individuals globally and representing one of the leading causes of hospitalization in both Europe and the United States [1, 2]. The global burden of heart failure is increasing by approximately 2 million new cases each year, and its 5-year survival rate is comparable to that of malignant tumors [3]. A growing body of evidence indicates that chronic hypertension can contribute to both macrovascular diseases (e.g., aortic dissection and heart failure) and microvascular complications (e.g., kidney disease and retinal damage) [4–6]. The coexistence of CHF and hypertension is associated with substantially worse clinical outcomes and a greater healthcare burden, emphasizing the pressing requirement to discover new clinical biomarkers for the early detection of CHF in hypertensive populations.

Inflammation and immune system activation are pivotal in the progression of cardiovascular diseases, particularly atherosclerosis [7] and heart failure [8]. Interleukin-6 (IL-6) and C-reactive protein (CRP), as representative circulating inflammatory mediators, have been demonstrated to correlate independently with greater cardiovascular risk and adverse clinical outcomes [9, 10]. Neutrophils contribute to the development and progression of atherosclerosis and cardiovascular disease by promoting inflammatory responses, oxidative stress, endothelial injury, and plaque instability [11]. In contrast, high-density lipoprotein (HDL) exerts protective cardiovascular effects through reverse cholesterol transport, as well as antioxidant, anti-inflammatory, and endothelial-protective properties [12]. Compared with individual biomarkers such as neutrophil count, neutrophil to lymphocyte ratio (NLR), or CRP, NHR integrates information on both pro-inflammatory activity and anti-inflammatory protection. It may thus serve as a complementary indicator reflecting the balance between inflammatory and anti-inflammatory processes and may offer insight in cardiovascular risk assessment [13, 14]. Previous studies have also observed associations between NHR and various disease conditions, including chronic kidney disease and periodontitis [15, 16].

Recent investigations have reported that NHR is linked to coronary artery plaque formation among individuals with coronary heart disease [14], and may be related to all-cause and cardiovascular mortality [17]. Nevertheless, the association of NHR with CHF has not been fully elucidated. Most existing evidence is derived from studies in the general population, while research focusing specifically on high-risk populations, such as individuals with hypertension, is limited. Hypertension is the most prevalent modifiable risk factor for heart failure (HF). Its prevalence among patients with HF increased from 66% in 1985–1990 to 74% in 1997–2002, highlighting its important contribution to the development of both heart failure with reduced ejection fraction (HFrEF) and heart failure with preserved ejection fraction (HFpEF) [18]. Hypertension may promote myocardial injury and cardiac remodeling through mechanisms including endothelial dysfunction, inflammatory activation, and overactivation of the renin-angiotensin system (RAS), potentially amplifying the pathological link between NHR and CHF [19]. Therefore, exploring the potential association between NHR and CHF in hypertensive populations may provide some theoretical insight and clinical relevance.

Machine learning (ML) models offer substantial advantages over traditional statistical methods in modeling high-dimensional data, nonlinear associations, and intricate feature interactions. These models have been successfully applied across diverse medical fields, including disease diagnosis, prognostic prediction, treatment optimization, and medical image analysis [20], and have demonstrated promising potential for improving the accuracy of CVD risk prediction and etiological assessment [21]. Given this background, the present study sought to explore the link between NHR levels and the prevalence of CHF in adults with hypertension, using data from the National Health and Nutrition Examination Survey (NHANES). In addition, ML techniques were adopted to establish and interpret a CHF risk assessment model based on NHR and other clinical variables. The study’s findings may contribute to improved approaches for identifying and managing high-risk individuals at an early stage.

## Methods

### Survey description

Our investigation is grounded in NHANES data, collected through a program conducted by the US National Center for Health Statistics (NCHS). By employing a multistage, stratified probability sampling design, NHANES ensures representation of the non-institutionalized civilian population across the United States [22]. Relevant datasets have been deposited and are freely available at https://www.cdc.gov/nchs/nhanes/.

### Sample under investigation

**Fig. 1:**
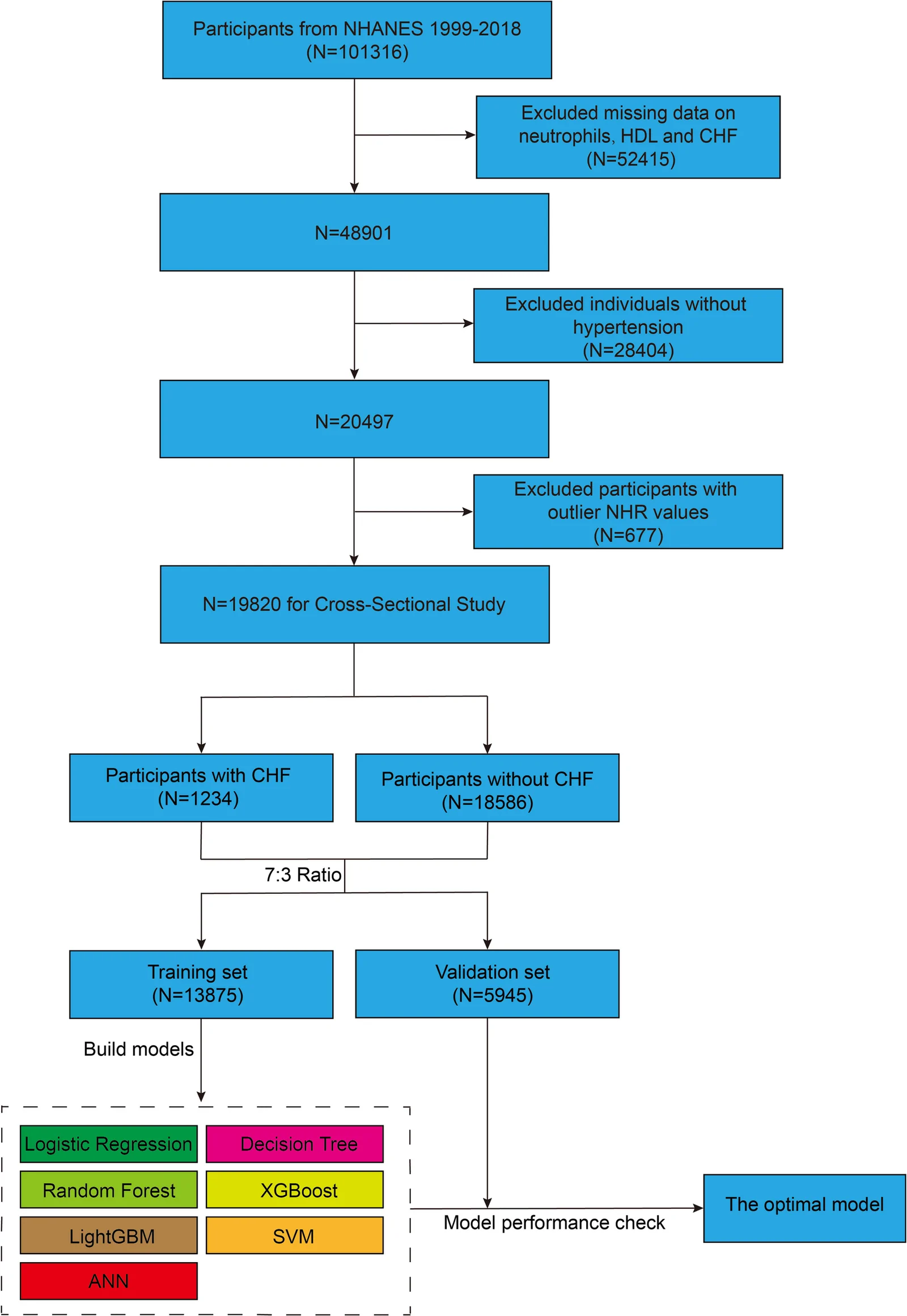
Flowchart of the sample selection from NHANES 1999-2018. NHANES, National Health and Nutrition Examination Survey; HDL, high-density lipoprotein cholesterol; CHF, congestive heart failure; NHR, neutrophil to high-density lipoprotein cholesterol ratio; LightGBM, Light Gradient Boosting Machine; XGBoost, Extreme Gradient Boosting; SVM, Support Vector Machine; ANN, Artificial Neural Network

This research utilized data from ten consecutive NHANES cycles spanning 1999 to 2018, encompassing demographic information, physical examination findings, laboratory results, and questionnaire responses. In the initial phase, 101,316 individuals were screened. Subjects were included based on the following requirements: age ≥ 20 years, availability of complete data on HDL-C, neutrophil count, and CHF, and fulfillment of at least one of the following hypertension diagnostic criteria: self-reported hypertension, ongoing antihypertensive treatment, an average diastolic blood pressure (DBP) ≥ 90 mmHg, or systolic blood pressure (SBP) ≥ 140 mmHg. Outliers in NHR were identified using the interquartile range (IQR) method. Values below Q1–1.5 × IQR or above Q3 + 1.5 × IQR were defined as abnormal values and excluded from the primary analysis. The final analytical sample comprised 19,820 hypertensive individuals with complete data **(**Fig. 1**)**.

### Definition of NHR and CHF

NHR was the primary exposure variable in this study, obtained by dividing *neutrophil count (10³ cells/µL)* by *HDL concentration (mmol/L)* [23]. NHR functions as a composite indicator reflecting the inflammatory activity and lipid metabolic processes. It was investigated as a continuous variable and subsequently classified into quartiles for descriptive statistics and regression modeling. The corresponding NHANES variable codes were LBDNENO (neutrophils) and LBDHDDSI/LBDHDLSI (HDL-C).

The primary outcome was CHF, defined as a self-reported diagnosis by a physician or other health professional, based on NHANES questionnaire data (variable code: MCQ160B).

### Selection of covariates

Covariates were chosen with reference to previous studies [17, 24] linking them to inflammation, CHF, and cardiovascular risk in hypertensive populations, as well as data availability within the NHANES database. Continuous covariates included: glycated hemoglobin A_1c_ (HbA_1c_, %), albumin (g/dL), fasting plasma glucose (FPG, mmol/L), estimated glomerular filtration rate (eGFR, mL/min/1.73 m²), aspartate aminotransferase (AST, U/L), serum creatinine (mg/dL), and alanine aminotransferase (ALT, U/L). Categorical covariates included: age (≥ 60 or < 60 years), PIR (< 1, 1-3.9, ≥ 4), sex, race (non-Hispanic Black, Mexican American, non-Hispanic White, other races, other Hispanic), education level (> high school, < high school, high school graduate), marital condition (widowed, divorced, never married, married), body mass index (BMI: ≥ 30, 25-29.9, 18.5–24.9, and < 18.5 kg/m²), history of smoking (lifetime smoking exposure of ≥ 100 cigarettes), drinking frequency (weekly, monthly, yearly), and history of diabetes and medication use (glucose-lowering, lipid-lowering, antihypertensive, and heart failure medications). The variance inflation factor (VIF) was used to assess multicollinearity among covariates in the logistic regression models. The diagnosis of diabetes was established if any of the following conditions were met: 2-hour oral glucose tolerance test (OGTT) ≥ 11.1 mmol/L, FPG ≥ 7.0 mmol/L, participant-reported clinician diagnosis, or treatment with glucose-lowering medications. All variables were obtained from the NHANES questionnaire, physical examination, laboratory, and demographic datasets, with measurements conducted using standardized procedures as described on the official NHANES webpage.

### Statistical analysis

All analyses accounted for the complex sampling design of NHANES by incorporating sampling weights, strata, and primary sampling units. For the combined survey cycles, 10-year sampling weights were calculated as 4-year weight × 2/10 and 2-year weight × 1/10. All descriptive and regression analyses were survey-weighted. All continuous variables were presented as survey-weighted means and standard deviations, while categorical variables were expressed as survey-weighted frequencies and percentages. Participants were categorized into quartiles according to NHR levels, and intergroup differences were assessed using survey-weighted chi-square tests for categorical variables and survey-weighted linear regression for continuous variables, accounting for the complex sampling design of NHANES.

The association between NHR and CHF was evaluated using a three-step survey-weighted multivariable logistic regression approach: In Model 1, no covariates were adjusted; Model 2 incorporated adjustments for race, sex, and age; and Model 3 was further adjusted for sociodemographic characteristics, behavioral factors, comorbidities, and laboratory markers based on Model 2. Nonlinear associations were evaluated using restricted cubic spline (RCS) curves. Stratified subgroup analyses were conducted to assess heterogeneity. To assess the robustness of the association between NHR and CHF, a series of sensitivity analyses were performed. First, the analyses were repeated in the overall adult population. Second, participants with previously excluded extreme NHR values were reintroduced to evaluate the potential impact of outlier handling. Third, alternative covariate adjustment strategies were applied: in model a, the four medication-use variables were removed and SBP and DBP were added; in model b, FPG, HbA_1c_, BMI, diabetes, smoking status, and drinking frequency were removed and triglycerides (TG) and total cholesterol (TC) were included. In addition, several categorical variables were redefined using alternative cutoff points, including age, marital status, BMI, and family PIR. Finally, an unweighted analysis was conducted and compared with the primary weighted analysis. The predictive performance of NHR and other inflammatory markers for CHF was evaluated by means of receiver operating characteristic (ROC) curves. The areas under the curve (AUCs) along with their 95% confidence intervals (CIs) were estimated. We carried out a mediation analysis to investigate the potential mediating effect of serum albumin within the correlation of NHR with CHF.

Furthermore, participants were randomly allocated into the training set and validation set in a 7:3 ratio. Variable selection was conducted using the Least Absolute Shrinkage and Selection Operator (LASSO) regression. Seven ML models were developed in the training set: LR, Random Forest (RF), Decision Tree (DT), Extreme Gradient Boosting (XGBoost), Light Gradient Boosting Machine (LightGBM), Support Vector Machine (SVM), and Artificial Neural Network (ANN). Five-fold cross-validation and grid search were applied for hyperparameter improvement. The model’s effectiveness was assessed by applying the AUC, specificity, sensitivity, and additional performance metrics. The discrimination and calibration capacities were measured with Receiver Operating Characteristic (ROC) curves and calibration curves, respectively, while clinical utility was estimated through decision curve analysis (DCA).

R (v4.5.1), Python (3.12.7), and Zstats (v0.90) served as the primary tools for data processing and statistical evaluation. Statistical significance was set at a two-tailed p-value of < 0.05.

## Results

### Baseline features of individuals

This research incorporated a total of 19,820 participants (Fig. 1), of whom 53.00% were aged ≥ 60 years, and 52.00% were male. Participants were stratified into quartiles based on NHR values, with mean ± standard deviations NHRs of 1.69 ± 0.41, 2.73 ± 0.26, 3.74 ± 0.34, and 5.59 ± 0.91 for the first to fourth quartiles, respectively. The overall prevalence of CHF was 4.90%. Baseline characteristics differed significantly across NHR quartiles for all variables except education level (all *p* < 0.05). Participants in the higher NHR quartiles were more likely to be aged ≥ 60 years, have diabetes, have smoked at least 100 cigarettes in their lifetime, report yearly alcohol consumption, and use glucose-lowering medications. In addition, levels of TG, FPG, HbA_1c_, ALT, serum creatinine, eGFR, lymphocyte-to-HDL ratio (LHR), systemic immune-inflammation index (SII), and platelet-to-HDL ratio (PHR) were significantly higher, while levels of HDL-C, TC, AST, and platelet-to-lymphocyte ratio (PLR) were lower (all *p* < 0.05). Increasing NHR levels were significantly correlated with higher CHF prevalence (*p* < 0.05) (Table 1). When comparing individuals by CHF status, those with CHF were more likely to be non-Hispanic White, have lower educational attainment, be unmarried, have lower family income, exhibit higher rates of smoking and diabetes, and report greater medication use. They also exhibited elevated levels of FPG, HbA_1c_, and serum creatinine, and reduced levels of HDL-C, TC, albumin, ALT, eGFR, and frequency of alcohol consumption (Table S1).

**Table 1:** Baseline characteristics of the study population according to NHR quartiles

Characteristics	Overall *N* = 72,910,237	Q1 *N* = 18,237,039	Q2 *N* = 18,289,988	Q3 *N* = 18,206,939	Q4 *N* = 18,176,271	*p*-value
Male, (%)	37,815,420 (52%)	12,041,653 (66%)	9,940,955 (54%)	8,604,147 (47%)	7,228,664 (40%)	< 0.001
Age ≥ 60, (%)	38,649,104 (53%)	8,657,069 (47%)	9,121,716 (50%)	9,838,059 (54%)	11,032,260 (61%)	< 0.001
Race, (%)						< 0.001
Mexican American	4,076,731 (5.6%)	636,391 (3.5%)	968,113 (5.3%)	1,204,780 (6.6%)	1,267,448 (7.0%)	
Other Hispanic	3,245,109 (4.5%)	646,442 (3.5%)	781,104 (4.3%)	948,144 (5.2%)	869,418 (4.8%)	
Non-Hispanic White	51,531,971 (71%)	11,675,926 (64%)	12,980,758 (71%)	13,270,310 (73%)	13,604,977 (75%)	
Non-Hispanic Black	9,627,638 (13%)	4,187,803 (23%)	2,406,096 (13%)	1,688,487 (9.3%)	1,345,252 (7.4%)	
Other Races	4,428,788 (6.1%)	1,090,477 (6.0%)	1,153,918 (6.3%)	1,095,218 (6.0%)	1,089,176 (6.0%)	
Education level, (%)						0.067
< high school	5,473,041 (7.5%)	1,170,205 (6.4%)	1,402,507 (7.7%)	1,419,355 (7.8%)	1,480,975 (8.1%)	
High school	8,971,687 (12%)	2,182,767 (12%)	2,216,733 (12%)	2,236,465 (12%)	2,335,722 (13%)	
> high school	58,465,509 (80%)	14,884,067 (82%)	14,670,748 (80%)	14,551,120 (80%)	14,359,574 (79%)	
Marital status, (%)						< 0.001
Married	46,378,747 (64%)	11,142,151 (61%)	11,536,617 (63%)	11,993,115 (66%)	11,706,864 (64%)	
Widowed	8,786,781 (12%)	2,503,842 (14%)	2,614,624 (14%)	2,106,932 (12%)	1,561,384 (8.6%)	
Divorced	10,629,956 (15%)	2,924,584 (16%)	2,527,614 (14%)	2,361,098 (13%)	2,816,659 (15%)	
Never married	7,114,754 (9.8%)	1,666,462 (9.1%)	1,611,133 (8.8%)	1,745,794 (9.6%)	2,091,364 (12%)	
Family PIR, (%)						< 0.001
Low income(< 1)	9,726,120 (13%)	2,069,931 (11%)	2,296,264 (13%)	2,510,562 (14%)	2,849,363 (16%)	
Median income(1-3.9)	37,139,981 (51%)	9,111,040 (50%)	9,408,660 (51%)	9,188,973 (50%)	9,431,308 (52%)	
High income(≥ 4)	26,044,136 (36%)	7,056,068 (39%)	6,585,065 (36%)	6,507,404 (36%)	5,895,599 (32%)	
BMI, (%)						< 0.001
< 18.5	34,314,961 (47%)	5,648,108 (31%)	8,078,931 (44%)	9,196,334 (51%)	11,391,588 (63%)	
18.5–24.9	24,326,896 (33%)	6,717,812 (37%)	6,535,766 (36%)	6,327,488 (35%)	4,745,830 (26%)	
25-29.9	13,490,651 (19%)	5,493,524 (30%)	3,492,539 (19%)	2,544,878 (14%)	1,959,710 (11%)	
≥ 30	777,729 (1.1%)	377,595 (2.1%)	182,753 (1.0%)	138,239 (0.8%)	79,142 (0.4%)	
Diabetes, (%)	16,983,220 (23%)	2,606,882 (14%)	3,830,519 (21%)	4,560,498 (25%)	5,985,321 (33%)	< 0.001
Smoked at least 100 cigarettes in life, (%)	36,234,894 (50%)	8,039,461 (44%)	8,667,683 (47%)	9,394,892 (52%)	10,132,857 (56%)	< 0.001
Drinking frequency, (%)						< 0.001
Weekly	30,714,470 (42%)	8,933,210 (49%)	7,979,432 (44%)	7,384,418 (41%)	6,417,409 (35%)	
Monthly	16,280,951 (22%)	3,729,349 (20%)	3,915,570 (21%)	4,226,982 (23%)	4,409,051 (24%)	
Yearly	25,914,816 (36%)	5,574,480 (31%)	6,394,986 (35%)	6,595,539 (36%)	7,349,811 (40%)	
The use of antihypertensive medications, (%)	50,125,823 (69%)	12,145,587 (67%)	12,479,304 (68%)	12,714,135 (70%)	12,786,797 (70%)	0.011
The use of lipid-lowering medications, (%)	26,469,538 (36%)	5,862,499 (32%)	6,654,888 (36%)	6,999,663 (38%)	6,952,488 (38%)	< 0.001
The use of glucose-lowering medications, (%)	11,034,005 (15%)	1,546,585 (8.5%)	2,428,059 (13%)	2,908,532 (16%)	4,150,829 (23%)	< 0.001
The use of heart failure medications, (%)	35,415,243 (49%)	8,421,279 (46%)	8,925,972 (49%)	8,923,215 (49%)	9,144,777 (50%)	0.023
Albumin (g/dL)	4.22± (0.34)	4.23± (0.33)	4.24± (0.33)	4.23± (0.34)	4.20± (0.36)	0.007
ALT (U/L)	26.29± (20.03)	24.27± (16.88)	25.48± (16.11)	26.91± (20.15)	28.50± (25.41)	< 0.001
AST (U/L)	26.21± (16.38)	27.26± (18.48)	25.78± (12.73)	26.23± (19.00)	25.58± (14.39)	< 0.001
Serum creatinine (mg/dL)	0.95± (0.53)	0.91± (0.52)	0.93± (0.47)	0.95± (0.49)	0.99± (0.61)	< 0.001
eGFR (ml/min/1.73 m²)	83.01± (22.34)	81.79± (20.85)	82.19± (21.64)	83.16± (22.48)	84.91± (24.15)	< 0.001
FPG (mmol/L)	6.30± (2.12)	5.85± (1.51)	6.18± (1.97)	6.36± (2.07)	6.82± (2.64)	< 0.001
HbA_1c_ (%)	5.84± (1.07)	5.62± (0.79)	5.80± (1.02)	5.88± (1.07)	6.08± (1.30)	< 0.001
HDL-C (mmol/L)	1.37± (0.43)	1.78± (0.47)	1.41± (0.31)	1.24± (0.27)	1.06± (0.23)	< 0.001
TG (mmol/L)	1.69± (1.22)	1.22± (0.80)	1.54± (0.97)	1.78± (1.09)	2.24± (1.63)	< 0.001
TC (mmol/L)	5.16± (1.11)	5.36± (1.07)	5.18± (1.05)	5.14± (1.15)	4.97± (1.12)	< 0.001
NHR	3.43± (1.54)	1.69± (0.41)	2.73± (0.26)	3.74± (0.34)	5.59± (0.91)	< 0.001
LHR	1.70± (1.36)	1.14± (0.90)	1.51± (0.63)	1.83± (1.19)	2.33± (2.00)	< 0.001
PHR	197.98± (76.49)	142.37± (50.16)	180.99± (56.88)	210.36± (65.53)	258.46± (78.65)	< 0.001
PLR	132.42± (55.65)	140.35± (57.66)	132.26± (52.24)	129.55± (55.28)	127.48± (56.44)	< 0.001
NLR	2.29± (1.19)	1.75± (0.84)	2.11± (0.94)	2.43± (1.18)	2.88± (1.41)	< 0.001
SII	573.00± (348.05)	414.57± (222.08)	512.36± (257.15)	608.11± (334.72)	757.83± (439.82)	< 0.001
Congestive Heart Failure, (%)	3,605,702 (4.9%)	587,968 (3.2%)	761,641 (4.2%)	984,462 (5.4%)	1,271,630 (7.0%)	< 0.001

All continuous variables were presented as survey-weighted means and standard deviations, while categorical variables were expressed as survey-weighted frequencies and percentages

*Abbreviations*: *NHR* Neutrophil to high-density lipoprotein cholesterol ratio, *Q* quartiles, *PIR* Poverty Income Ratio, *BMI* Body Mass Index, *HDL-C* high-density lipoprotein cholesterol, *TG* triglycerides, *TC* total cholesterol, *FPG* fasting plasma glucose, *HbA*_1c_ glycated hemoglobin A1c, *ALT* alanine aminotransferase, *AST* aspartate aminotransferase, *eGFR* estimated glomerular filtration rate, *LHR* lymphocyte to high-density lipoprotein ratio, *PHR* platelet to high-density lipoprotein ratio, *PLR* platelet to lymphocyte ratio, *NLR* Neutrophil to lymphocyte ratio, *SII* systemic immune-inflammation index

*P* values were calculated using survey-weighted chi-square tests for categorical variables and survey-weighted linear regression for continuous variables

### Connection between NHR and prevalence of CHF

**Fig. 2:**
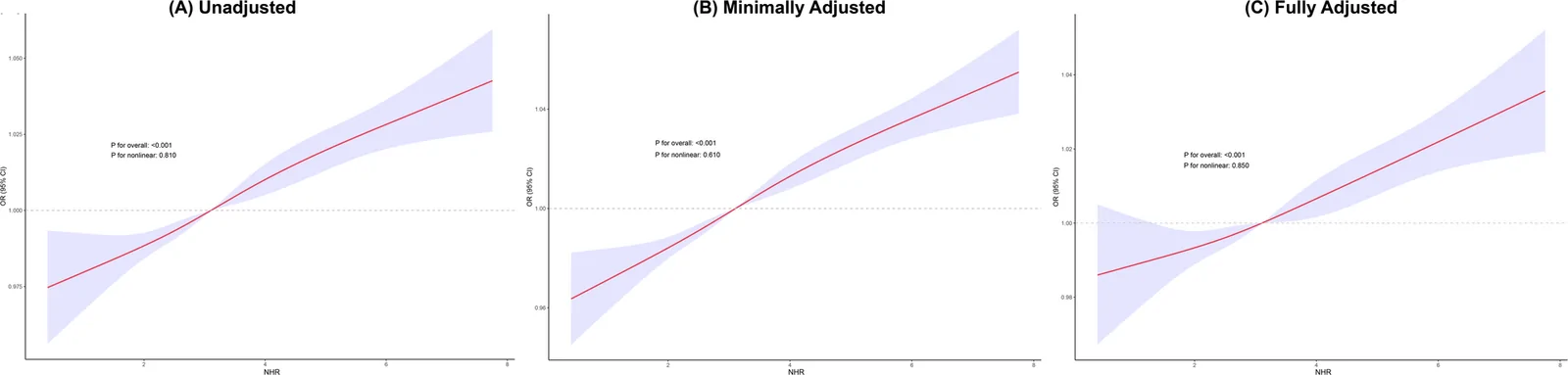
RCS analyses of the relationship between NHR and CHF. **A** Unadjusted model. **B** Model adjusted with age, sex, and race. **C** Model adjusted with age, sex, race, education level, marital status, family PIR, BMI, smoked at least 100 cigarettes in life, drinking frequency, diabetes, the use of glucose-lowering medications, lipid-lowering medications, antihypertensive medications, and heart failure medications, FPG, HbA_1c_, albumin, ALT, AST, serum creatinine, and eGFR. RCS, restricted cubic spline; NHR, neutrophil to high-density lipoprotein cholesterol ratio; CHF, congestive heart failure; OR, odds ratio; CI, confidence interval

All included covariates had VIF values below 5 (Table S2). NHR was considerably and positively correlated with the prevalence of CHF. Even after adjusting for potential confounders, a one-unit increase in NHR was linked to a 15% elevated prevalence of CHF (odds ratio [OR] = 1.15, 95% confidence interval [CI]: 1.10–1.21). When NHR was evaluated categorically by quartiles, the association remained robust. CHF prevalence was elevated by 69% in participants classified in the highest NHR quartile versus those in the lowest (OR = 1.69, 95% CI: 1.34–2.13; p for trend < 0.01) (Table 2). In the fully adjusted model, the RCS curve revealed a stable linear increasing trend between NHR and CHF prevalence (Fig. 2).

**Table 2:** Associations between NHR and CHF

NHR	CHF		
	OR	95% CI	*P*
Crude model (Model 1)			
Continuous	1.21	1.16–1.27	< 0.001
Categories			
Quartile 1	1 (ref)		
Quartile 2	1.30	1.05–1.61	0.015
Quartile 3	1.72	1.41–2.09	< 0.001
Quartile 4	2.26	1.84–2.78	< 0.001
*P* for tend	1.84	1.59–2.12	< 0.001
Minimally adjusted model (Model 2)			
Continuous	1.31	1.25–1.37	< 0.001
Categories			
Quartile 1	1 (ref)		
Quartile 2	1.44	1.15–1.79	0.001
Quartile 3	2.05	1.67–2.53	< 0.001
Quartile 4	3.02	2.42–3.77	< 0.001
*P* for tend	2.27	1.95–2.65	< 0.001
Fully adjusted model (Model 3)			
Continuous	1.15	1.10–1.21	< 0.001
Categories			
Quartile 1	1 (ref)		
Quartile 2	1.11	0.88–1.41	0.400
Quartile 3	1.44	1.15–1.79	0.002
Quartile 4	1.69	1.34–2.13	< 0.001
*P* for tend	1.51	1.28–1.77	< 0.001

The neutrophil to high-density lipoprotein cholesterol ratio was converted from a continuous variable to a categorical variable (quartiles). All regression coefficients, ORs, and 95% CIs are weighted estimates based on survey-weighted logistic regression

Model 1: no covariates were adjusted

Model 2: age, sex, and race were adjusted

Model 3: age, sex, race, education level, marital status, family PIR, BMI, smoked at least 100 cigarettes in life, drinking frequency, diabetes, the use of glucose-lowering medications, lipid-lowering medications, antihypertensive medications, and heart failure medications, FPG, HbA_1c_, ALB, ALT, AST, serum creatinine, and eGFR were adjusted

*Abbreviations*: *OR* odds ratio, *95% CI* 95% confidence interval, *NHR* neutrophil to high-density lipoprotein cholesterol ratio, *CHF* Congestive Heart Failure

### Stratified analyses and sensitivity analysis

**Fig. 3:**
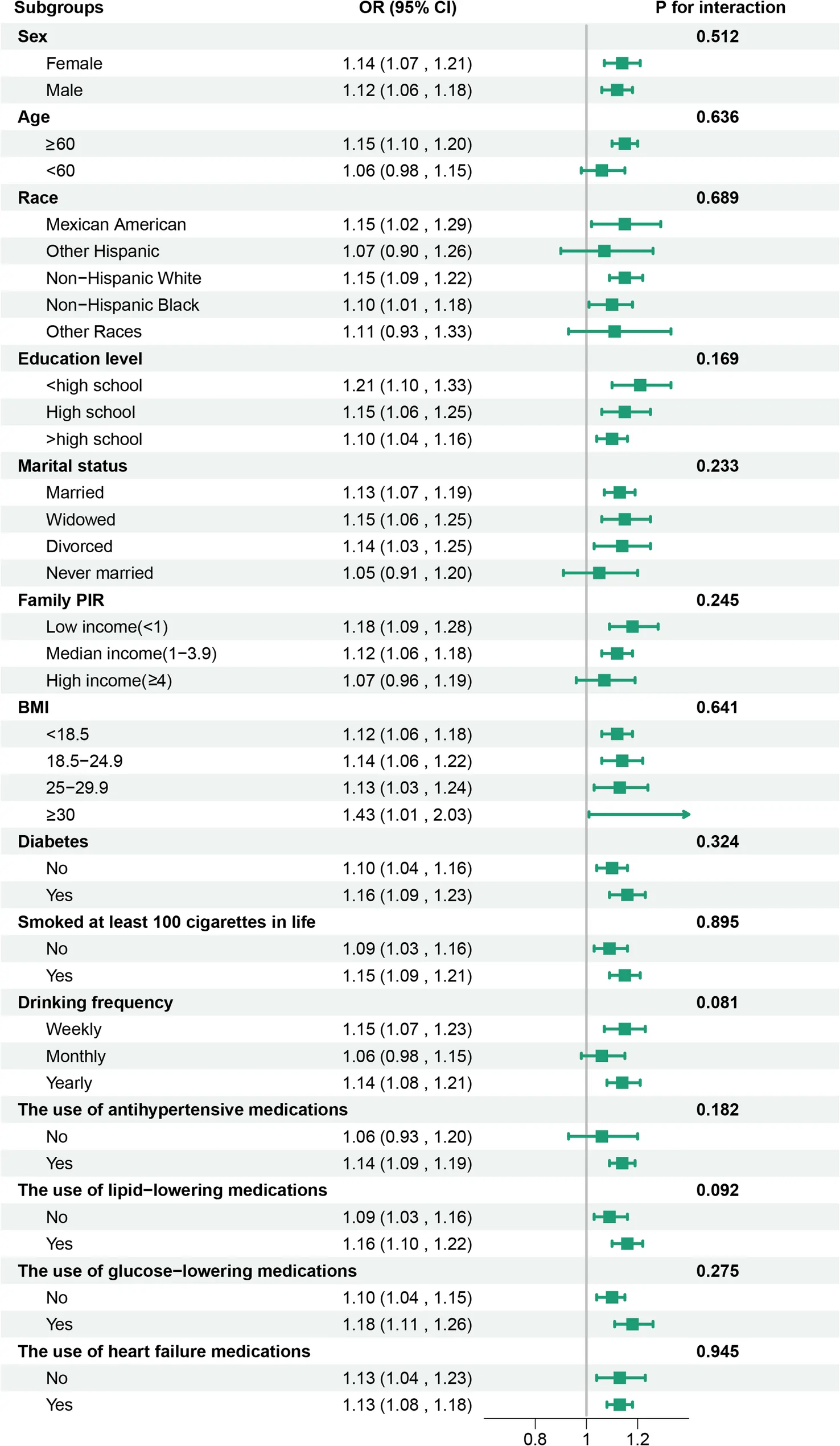
Subgroup analyses of the correlation between NHR and CHF. The odds ratios presented were derived from regression models in which NHR was entered as a continuous variable. Age, sex, race, education level, marital status, family PIR, BMI, smoked at least 100 cigarettes in life, drinking frequency, diabetes, the use of glucose-lowering medications, lipid-lowering medications, antihypertensive medications, and heart failure medications, FPG, HbA_1c_, albumin, ALT, AST, serum creatinine, and eGFR were adjusted. OR, odds ratio; CI, confidence interval; NHR, neutrophil to high-density lipoprotein cholesterol ratio; CHF, congestive heart failure; BMI, body mass index; PIR, poverty income ratio

To assess the robustness of the NHR-CHF relationship, subgroup analyses were performed using fully adjusted logistic regression models across various sociodemographic and clinical subgroups **(**Fig. 3**)**. The association remained consistent in all subgroups, and no significant interactions were observed (all interaction p-values > 0.05). In sex-stratified analyses, similar associations were observed in both male (OR = 1.12) and female (OR = 1.14) (interaction *p* = 0.512). Comparable trends were also noted across different levels of educational attainment (interaction *p* = 0.169). Moreover, no significant effect modification was found by marital status, smoking status, age, family PIR, race, BMI, drinking frequency, or diabetes status, suggesting that the association between NHR and CHF is broadly applicable across diverse population groups.

Across all sensitivity analyses, the association between NHR and CHF remained broadly consistent in both direction and strength. The findings from logistic regression and restricted cubic spline analyses were largely unchanged across analyses of the overall population, analyses including outliers, models with different combinations of covariate adjustment, models using alternative cutoff points for categorical variables, and unweighted analyses, further supporting the robustness of the observed association (Table S5-11, Figure S2-6).

### ROC curve analysis and mediation effect analysis

An AUC of 0.588 was obtained for NHR in discriminating CHF based on ROC analysis, which was higher than those of other inflammatory markers, including SII (AUC = 0.533), LHR (AUC = 0.519), PHR (AUC = 0.519), and PLR (AUC = 0.499), but lower than that of NLR (AUC = 0.601). DeLong’s test showed that NHR performed significantly better than LHR, PHR, SII, and PLR (all *P* < 0.001), whereas its difference from NLR was not statistically significant (*P* = 0.162) (Table S3, Figure S1). These findings suggest that NHR showed only limited additional discriminatory value compared with the other inflammatory markers evaluated.

**Fig. 4:**
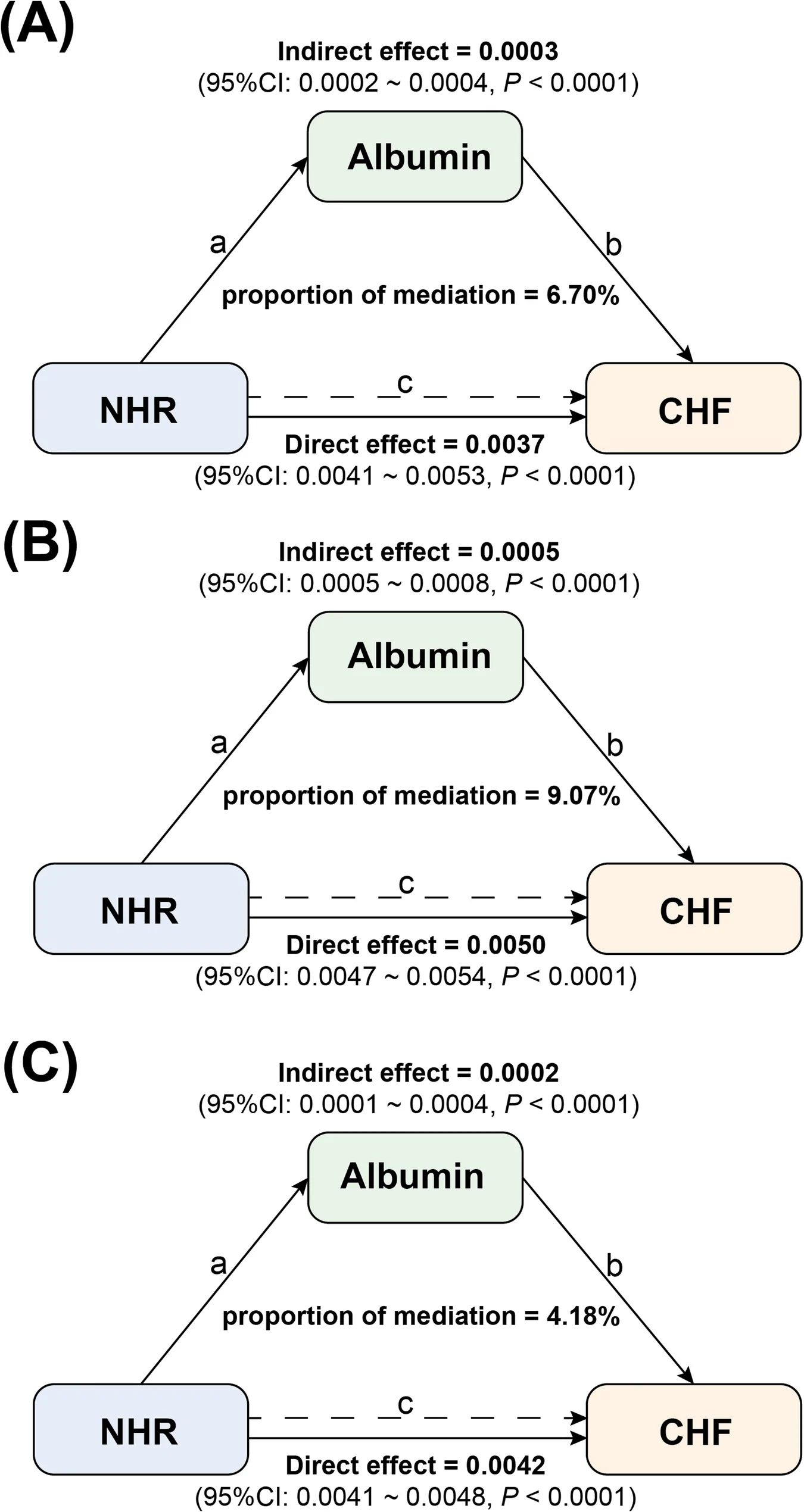
Estimated proportion of the association between NHR and CHF by albumin. **A** Unadjusted model. **B** Model adjusted with age, sex, and race. **C** Model adjusted with age, sex, race, education level, marital status, family PIR, BMI, smoked at least 100 cigarettes in life, drinking frequency, diabetes, the use of glucose-lowering medications, lipid-lowering medications, antihypertensive medications, and heart failure medications, FPG, HbA_1c_, albumin, ALT, AST, serum creatinine, and eGFR. NHR, neutrophil to high-density lipoprotein cholesterol ratio; CHF, congestive heart failure; CI, confidence interval

Furthermore, in the fully adjusted model, albumin was identified as a partial mediator in the NHR-CHF association, with a mediation proportion of 4.18% **(**Table 3; Fig. 4**)**, suggesting that the association between NHR and CHF prevalence may be partially explained by albumin levels.

**Table 3:** ALB mediated the association between the NHR and CHF

	ACME		ADE		Total effect		Proportion mediated
	Estimate	*P* value	Estimate	*P* value	Estimate	*P* value	
Model1	0.0003 (0.0002–0.0004)	< 0.0001	0.0037 (0.0041–0.0053)	< 0.0001	0.0040 (0.0045–0.0056)	< 0.0001	6.70%
Model2	0.0005 (0.0005–0.0008)	< 0.0001	0.0050 (0.0047–0.0054)	< 0.0001	0.0056 (0.0054–0.0063)	< 0.0001	9.07%
Model3	0.0002 (0.0001–0.0004)	< 0.0001	0.0042 (0.0041–0.0048)	< 0.0001	0.0045 (0.0033–0.0051)	< 0.0001	4.18%

Model 1: no covariates were adjusted

Model 2: age, sex, and race were adjusted

Model 3: age, sex, race, education level, marital status, family PIR, BMI, smoked at least 100 cigarettes in life, drinking frequency, diabetes, the use of glucose-lowering medications, lipid-lowering medications, antihypertensive medications, and heart failure medications, FPG, HbA_1c_, ALB, ALT, AST, serum creatinine, and eGFR were adjusted

*Abbreviations*: *ACME* average causal mediation effect, *ADE* average direct effect, *ALB* albumin, *NHR* neutrophil to high-density lipoprotein cholesterol ratio, *CHF* Congestive Heart Failure

### Construction and evaluation of the CHF ML models

**Fig. 5:**
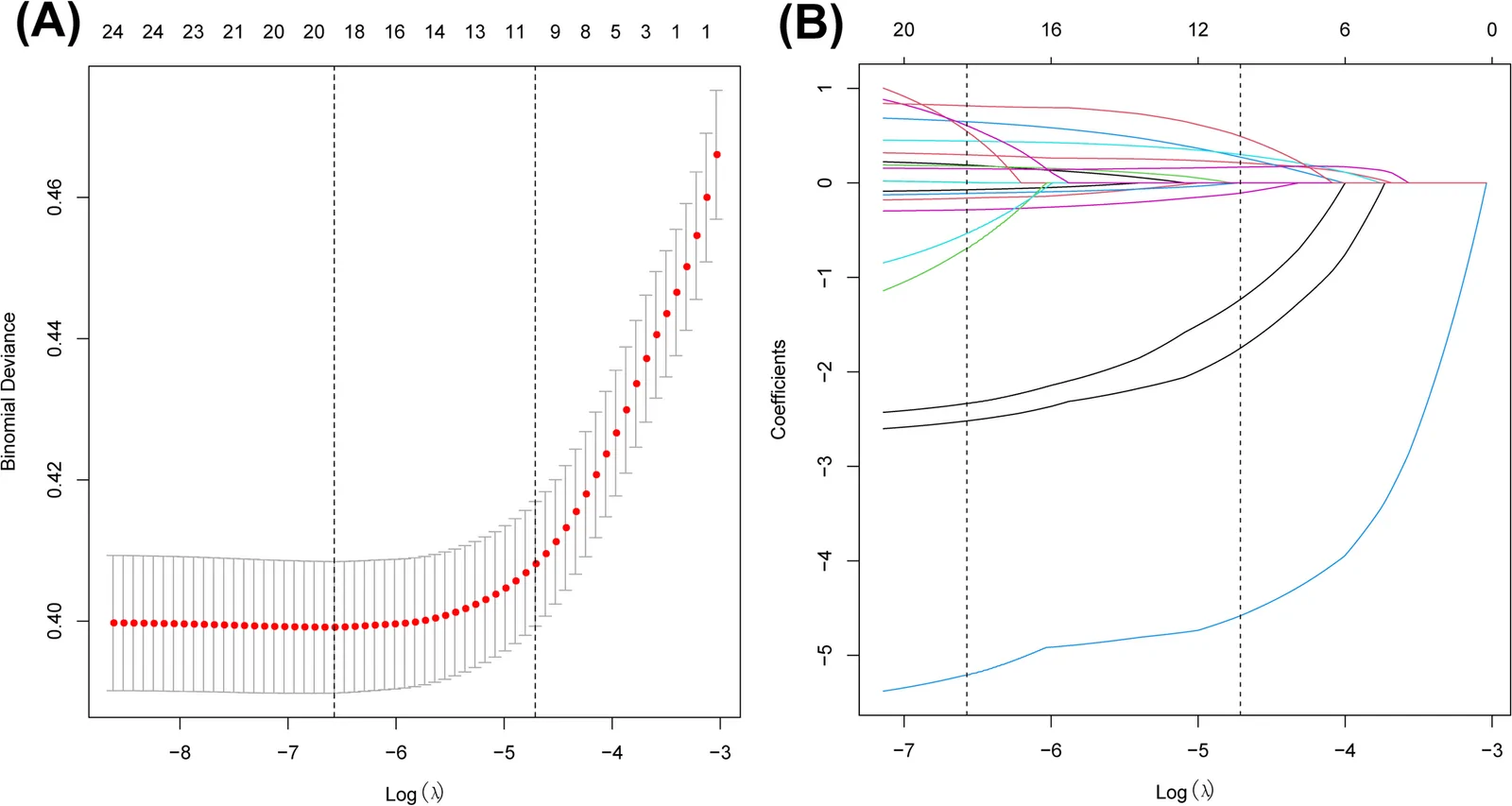
**A** 10-fold cross-validation for LASSO regression. **B** LASSO coefficient shrinkage path. LASSO, least absolute shrinkage and selection operator

**Fig. 6:**
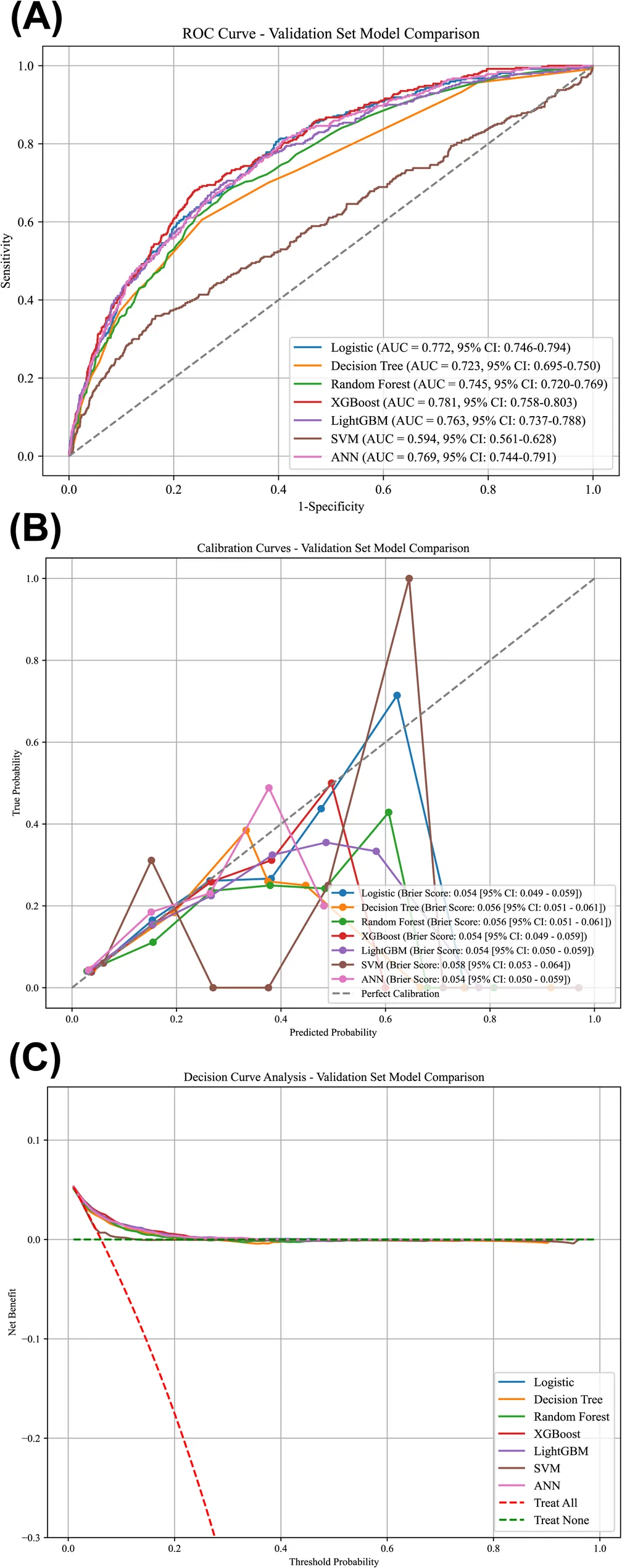
Predictive performance of seven machine learning models in the validation set. **A** ROC curves, **B** calibration curves, and (**C**) DCA curves. ROC, Receiver Operating Characteristic; DCA, Decision Curve Analysis; XGBoost, eXtreme Gradient Boosting; LightGBM, Light Gradient Boosting Machine; SVM, Support Vector Machine; ANN, Artificial Neural Network; AUC, Area Under the Curve; CI, confidence interval

Variable selection was conducted exclusively in the training set. LASSO regression was applied employing ten-fold cross-validation with the 1-SE rule (λ = 0.009, logλ = -4.713), resulting in 9 retained variables: albumin, eGFR, TC, NHR, family PIR, diabetes, the use of lipid-lowering, glucose-lowering, and antihypertensive medications (Fig. 5). All models except SVM attained AUC values > 0.700 in the validation set. XGBoost demonstrated the optimal overall effectiveness, with an AUC of 0.781 (95% CI: 0.758–0.803), the highest Kappa coefficient (0.234), PPV (0.268), and the lowest Brier score (0.054, 95% CI: 0.049–0.059). DCA showed that all models provided meaningful net clinical benefit across a wide range of threshold probabilities (Table 4; Fig. 6). LR (AUC = 0.772), ANN (AUC = 0.769), and LightGBM (AUC = 0.763) also exhibited strong predictive performance. Based on these results, XGBoost was selected as the optimal model for subsequent interpretation analyses. The detailed optimal hyperparameter configurations of multiple ML models are presented in Table S4.

**Table 4:** Predictive performance of machine learning models in the validation set

Model	AUC (95% CI)	Accuracy	Sensitivity	Specificity	Kappa	Youden index	PPV	NPV
LR	0.772 (0.746–0.794)	0.906	0.276	0.948	0.217	0.223	0.26	0.952
DT	0.723 (0.695–0.750)	0.885	0.305	0.923	0.188	0.229	0.209	0.952
RF	0.745 (0.720–0.769)	0.882	0.311	0.92	0.185	0.23	0.204	0.953
XGBoost	0.781 (0.758–0.803)	0.905	0.303	0.945	0.234	0.248	0.268	0.953
LightGBM	0.763 (0.737–0.788)	0.901	0.289	0.941	0.213	0.231	0.247	0.952
SVM	0.594 (0.561–0.628)	0.934	0.008	0.996	0.006	0.004	0.107	0.938
ANN	0.769 (0.744–0.791)	0.915	0.192	0.963	0.176	0.155	0.257	0.947

*Abbreviations*: *AUC* Area Under the Curve, *95% CI* 95% Confidence Interval, *PPV* Positive Predictive Value, *NPV* Negative Predictive Value, *LR* Logistic Regression, *DT* Decision Tree, *RF* Random Forest, *XGBoost* Extreme Gradient Boosting, *LightGBM* Light Gradient Boosting Machine, *SVM* Support Vector Machine, *ANN* Artificial Neural Network

## Discussion

The cross-sectional investigation, based on NHANES from 1999 to 2018, included a total of 19,820 participants. Elevated NHR was found to be significantly linked to an increased prevalence of CHF. The relationship persisted consistently and stably within various sociodemographic and clinical subgroups (all interaction p-values > 0.05). The mediation model suggested that albumin accounted in part for the relationship between NHR and CHF. Collectively, these findings suggest that NHR is associated with CHF prevalence in hypertensive populations and may serve as a supplementary marker for risk stratification.

Although numerous studies have examined the role of inflammation in cardiovascular disease, the predictive role of NHR in CHF, particularly among hypertensive populations, remains insufficiently understood. Verma et al. identified a notable relationship between serum CRP and heart failure symptoms among individuals with HFpEF across 18 countries, underscoring the central role of inflammation in heart failure pathophysiology [25]. Held et al. carried out a multinational, multicenter prospective study encompassing 15,828 subjects with stable coronary artery disease and found that elevated IL-6 levels independently predicted cardiovascular death, myocardial infarction, and hospitalization for heart failure [26]. In a large-scale cohort study in China, Liu et al. also detected that elevated NHR was strongly related to stroke and myocardial infarction, particularly among individuals with dysglycemia [27]. Chuang et al. reported that NHR outperformed the NLR in projecting cardiovascular disease risk in a community-based Taiwanese population [28]. In our study, NLR was also significantly associated with CHF, but its OR was lower than that of NHR. Although its univariable AUC was slightly higher than that of NHR, the difference was not statistically significant (Table S3, S12, and Figure S1, S7). Among these biomarkers, CRP is a classic acute-phase protein synthesized mainly by the liver in response to pro-inflammatory cytokines, especially IL-6, and is widely used as a marker of systemic inflammation [29]. IL-6 is an early inflammatory cytokine that stimulates hepatic production of acute-phase proteins such as CRP and contributes to the amplification of inflammatory signaling [30]. NLR mainly reflects the balance between pro-inflammatory neutrophils and lymphocyte-mediated immune regulation and is a widely used marker of systemic inflammatory status [31]. In contrast, NHR combines neutrophil-mediated pro-inflammatory activity with HDL-mediated anti-inflammatory and antioxidant effects, capturing a broader inflammatory-metabolic balance. While CRP, IL-6, and NLR primarily indicates systemic inflammatory status, NHR additionally reflects the protective role of lipid metabolism, providing complementary insight into inflammatory-metabolic interactions [32]. Regarding clinical applicability, NLR is a more established and widely used inflammatory marker that can be easily calculated from routine blood cell counts. In comparison, NHR requires both neutrophil counts and serum HDL levels; however, these parameters are also routinely measured in clinical practice, making NHR similarly convenient and cost-effective [17]. Compared with NLR, NHR may therefore offer a more biologically interpretable index by linking inflammatory activation with lipid-related protective mechanisms, rather than reflecting inflammation alone [33–35]. Therefore, the potential clinical value of NHR may lie more in its biological interpretability than in a substantial improvement in discriminatory performance. Previous studies have observed that NHR is elevated in specific disease conditions and may differ from other inflammatory markers. For example, in a cross-sectional study of women with gestational hypertension and preeclampsia, NHR levels were higher in the patient groups compared with normotensive controls, while other markers such as NLR, PLR, MLR, and CRP/albumin ratio did not show statistically significant differences [36]. Similarly, in patients with Parkinson’s disease, NHR levels were elevated relative to healthy controls, whereas IL-6 and SII were not significantly different [37].

The potential mechanisms underlying the observed positive NHR-CHF relationship remain incompletely understood. A leading hypothesis suggests that systemic inflammation in heart failure contributes to vascular injury and endothelial dysfunction, which in turn promotes coronary microvascular inflammation, myocardial hypertrophy, and diastolic dysfunction. Simultaneously, local inflammatory responses reduce nitric oxide (NO) bioavailability, inhibit protein kinase G (PKG) activity, and increase myocardial stiffness [38]. Chronic immune activation, featuring higher levels of proinflammatory cytokines, for example IL-6, IL-1β, and tumor necrosis factor-alpha (TNF-α), can exacerbate vascular remodeling and plaque instability, thereby increasing the probability of major adverse cardiovascular events (MACE) and thrombosis [39, 40]. In CHF, immune responses encompassing both adaptive and innate pathways are activated, involving macrophages, mast cells, B lymphocytes, and T lymphocytes. Macrophages release IL-10, which stimulates fibroblast activation and contributes to myocardial fibrosis; meanwhile, the toll-like receptor 4 (TLR4)-mediated innate immune pathway promotes the release of inflammatory mediators and pathological ventricular remodeling [41, 42]. Immune cell infiltration into cardiovascular tissues further exacerbates inflammation through the release of proinflammatory cytokines, induction of reactive oxygen species (ROS), activation of matrix metalloproteinases, and promotion of fibroblast proliferation, ultimately leading to interstitial fibrosis [43, 44]. Collectively, these mechanisms underscore the central contribution of inflammation to CHF development and offer a plausible explanation for the observed association between elevated NHR and increased CHF prevalence.

Further mediation analysis indicated that albumin plays a partial mediating role in the NHR-CHF relationship. Inflammatory states increase capillary permeability, leading to albumin extravasation and reduced serum albumin levels [45]. Hypoalbuminemia can exacerbate CHF progression through multiple mechanisms, including diminished oncotic pressure, diuretic resistance, worsened volume overload and malnutrition, and impaired renal function [46]. Older adults, in particular, may experience elevated pulmonary capillary filtration pressures in the setting of hypoalbuminemia due to decreased diastolic reserve and increased vascular stiffness, thereby increasing the risk of CHF [47]. Moreover, as an antioxidant and anti-inflammatory protein, reduced albumin levels may enhance oxidative stress, elevate blood viscosity, promote thrombogenesis, and aggravate cardiovascular damage [48]. These findings suggest that targeting albumin regulation may offer novel strategies for preventing and managing NHR-associated CHF. Clinically, albumin could be incorporated into routine evaluations and monitored alongside B-type natriuretic peptide (BNP) to enable earlier identification and intervention in patients at risk for CHF. In addition, because NHR is derived from routine blood count and lipid measurements and is readily available at low cost, it may still serve as an adjunctive marker in clinical practice when interpreted alongside established biomarkers such as BNP for the comprehensive assessment of CHF. Specifically, NHR may reflect inflammatory and metabolic status to some extent, whereas BNP is primarily used to indicate cardiac load and functional impairment; thus, the two markers provide information with different emphases. Therefore, in the clinical evaluation of hypertensive individuals, NHR may be considered as supplementary information based on routine laboratory testing and interpreted together with BNP and other conventional risk factors to help identify individuals who may be at risk of CHF or who may benefit from further cardiac functional assessment.

Our study extends previous findings by integrating ML approaches into the evaluation of the correlation between NHR and CHF prevalence. Compared with traditional statistical models, ML not only improved predictive performance but also enabled a more comprehensive assessment of complex nonlinear relationships and high-order interactions among clinical variables [49]. Among the seven algorithms, XGBoost demonstrated the best overall performance in the validation set, with superior discrimination, calibration, and classification metrics, and showed consistent stability in the training set. The favorable performance of XGBoost may be attributed to its strong regularization capacity, efficient computation, and ability to handle heterogeneous data structures and nonlinear patterns [50]. These characteristics have supported its widespread use in medical research. Previous studies have similarly reported the superiority of XGBoost in cardiovascular risk prediction, including models for short-term MACE after carotid endarterectomy and endovascular aneurysm repair [51, 52]. Together with our findings, these results highlight the robustness, generalizability, and clinical applicability of XGBoost-based models for cardiovascular risk stratification.

The present work is marked by several notable strengths. First, it is based on data derived from the NHANES, a nationally representative and demographically diverse dataset, which may provide some enhancement to the external validity and generalizability of the observations. Second, the enrollment of a wide range of sociodemographic, lifestyle, and metabolic covariates allowed for effective control of potential confounding factors. Third, the use of RCS curves offered insights into the potential nonlinear relationship between NHR and CHF. And the observed association remained consistent across multiple subgroups, supporting the broad applicability of the findings across diverse populations. Lastly, this study incorporated machine learning models for CHF risk prediction, providing additional methodological support for early risk stratification and identification of CHF in hypertensive populations. However, this study also has several limitations. First, this study used a cross-sectional design, with NHR and CHF assessed simultaneously; therefore, the temporal sequence could not be established, precluding any causal inference and leaving the possibility of reverse causation. Second, the CHF outcome was primarily based on self-reported physician diagnosis, which may be subject to recall bias and misclassification, and did not allow differentiation between heart failure subtypes. Although NT-proBNP data were available in some NHANES cycles, the sample coverage was limited, and follow-up data were unavailable; therefore, these data were not included in the primary analysis. Third, despite adjustment for multiple potential confounders, residual confounding from unmeasured factors may still exist, particularly because NHANES lacks information on some important variables, such as medication adherence and duration of hypertension. Fourth, the mediating proportion of albumin in the association between NHR and CHF was relatively low (4.18%), suggesting a limited mediating role. In addition, albumin is influenced by multiple factors, including nutritional status, hepatic synthesis, and volume status. Moreover, because albumin was measured only once in this study, its long-term mediating effect may have been underestimated. Fifth, this study primarily provides epidemiological evidence of an association and cannot elucidate the specific molecular or physiological mechanisms underlying the relationship between NHR and CHF. Furthermore, our ML models were developed and validated solely using the NHANES dataset with internal random splitting (7:3), without external cohort validation. Therefore, the generalizability of the models to other populations or clinical settings remains uncertain. Future studies are warranted to validate these findings in independent, prospective cohorts to assess the robustness and clinical applicability of the predictive models. Finally, although our study demonstrated an independent association between NHR and CHF, the incremental clinical advantage of NHR appears limited. Its clinical advantage over traditional inflammatory markers, such as NLR, SII, PLR, and LHR, remains uncertain. Therefore, the clinical utility of NHR as an additional predictive marker may be limited, and its value should be interpreted mainly as an associated biomarker rather than a strong tool for improving risk prediction. NHR may, to some extent, reflect the combined effects of neutrophil-mediated pro-inflammatory activity and HDL-mediated anti-inflammatory and antioxidant functions, providing complementary insight into inflammatory-metabolic balance. However, these findings should be further evaluated in future large-scale, prospective, and externally validated studies to clarify the clinical utility and incremental prognostic value of NHR.

## Conclusion

This study found an association between elevated NHR and CHF prevalence among hypertensive adults. These findings suggest that NHR may be a relevant biomarker in this population. However, further prospective studies are warranted to establish causality and to elucidate the underlying pathophysiological mechanisms.

## Supplementary Information

Supplementary Material 1: Figure S1. ROC curves for predicting CHF using inflammatory markers. ROC, Receiver Operating Characteristic; AUC, Area Under the Curve; NHR, neutrophil to high-density lipoprotein cholesterol ratio; CHF, congestive heart failure; SII, systemic immune-inflammation index; LHR, lymphocyte to high-density lipoprotein ratio; PHR, platelet to high-density lipoprotein ratio; PLR, platelet to lymphocyte ratio; NLR, neutrophil-to-lymphocyte ratio.

Supplementary Material 2: Figure S2. RCS analyses of the relationship between NHR and CHF in the overall population. (A) Unadjusted model. (B) Model adjusted with age, sex, and race. (C) Model adjusted with age, sex, race, education level, marital status, family PIR, BMI, smoked at least 100 cigarettes in life, drinking frequency, diabetes, the use of glucose-lowering medications, lipid-lowering medications, antihypertensive medications, and heart failure medications, FPG, HbA1c, albumin, ALT, AST, serum creatinine, and eGFR. RCS, restricted cubic spline; NHR, neutrophil to high-density lipoprotein cholesterol ratio; CHF, congestive heart failure; OR, odds ratio; CI, confidence interval.

Supplementary Material 3: Figure S3. RCS analyses of the relationship between NHR and CHF with outliers included. (A) Unadjusted model. (B) Model adjusted with age, sex, and race. (C) Model adjusted with age, sex, race, education level, marital status, family PIR, BMI, smoked at least 100 cigarettes in life, drinking frequency, diabetes, the use of glucose-lowering medications, lipid-lowering medications, antihypertensive medications, and heart failure medications, FPG, HbA1c, albumin, ALT, AST, serum creatinine, and eGFR. RCS, restricted cubic spline; NHR, neutrophil to high-density lipoprotein cholesterol ratio; CHF, congestive heart failure; OR, odds ratio; CI, confidence interval.

Supplementary Material 4: Figure S4. RCS analyses of the relationship between NHR and CHF. (A)(D) Unadjusted model. (B)(E) Model adjusted with age, sex, and race. (C) Model adjusted with age, sex, race, education level, marital status, family PIR, BMI, smoked at least 100 cigarettes in life, drinking frequency, diabetes, SBP, DBP, FPG, HbA1c, albumin, ALT, AST, serum creatinine, and eGFR. (F) Model adjusted with age, sex, race, education level, marital status, family PIR, the use of glucose-lowering medications, lipid-lowering medications, antihypertensive medications, and heart failure medications, TG, TC, albumin, ALT, AST, serum creatinine, and eGFR. RCS, restricted cubic spline; NHR, neutrophil to high-density lipoprotein cholesterol ratio; CHF, congestive heart failure; OR, odds ratio; CI, confidence interval.

Supplementary Material 5: Figure S5. RCS analyses of the relationship between NHR and CHF. (A)(D) Unadjusted model. (B)(E) Model adjusted with age, sex and race. (C)(F) Model adjusted with age, sex, race, education level, marital status, family PIR, BMI, smoked at least 100 cigarettes in life, drinking frequency, diabetes, the use of glucose-lowering medications, lipid-lowering medications, antihypertensive medications, and heart failure medications, FPG, HbA1c, albumin, ALT, AST, serum creatinine, and eGFR. (A)(B)(C) Age: 18-39 years→young group; 40-59 years→middle-aged group; ≥60 years→older group. Marital status: married; single. (D)(E)(F) PIR: < 1, very low income; 1-1.99, low income; 2-3.99, middle income; ≥ 4, high income. BMI: 18.5-24.9, normal; < 18.5 or ≥ 25, abnormal. RCS, restricted cubic spline; NHR, neutrophil to high-density lipoprotein cholesterol ratio; CHF, congestive heart failure; OR, odds ratio; CI, confidence interval.

Supplementary Material 6: Figure S6. RCS analyses of the relationship between NHR and CHF based on unweighted analyses. (A) Unadjusted model. (B) Model adjusted with age, sex, and race. (C) Model adjusted with age, sex, race, education level, marital status, family PIR, BMI, smoked at least 100 cigarettes in life, drinking frequency, diabetes, the use of glucose-lowering medications, lipid-lowering medications, antihypertensive medications, and heart failure medications, FPG, HbA1c, albumin, ALT, AST, serum creatinine, and eGFR. RCS, restricted cubic spline; NHR, neutrophil to high-density lipoprotein cholesterol ratio; CHF, congestive heart failure; OR, odds ratio; CI, confidence interval.

Supplementary Material 7: Figure S7. RCS analyses of the relationship between NLR and CHF. (A) Unadjusted model. (B) Model adjusted with age, sex, and race. (C) Model adjusted with age, sex, race, education level, marital status, family PIR, BMI, smoked at least 100 cigarettes in life, drinking frequency, diabetes, the use of glucose-lowering medications, lipid-lowering medications, antihypertensive medications, and heart failure medications, FPG, HbA1c, albumin, ALT, AST, serum creatinine, and eGFR. RCS, restricted cubic spline; NHR, neutrophil to high-density lipoprotein cholesterol ratio; CHF, congestive heart failure; OR, odds ratio; CI, confidence interval.

Supplementary Material 8.

Supplementary Material 9.

Supplementary Material 10.

Supplementary Material 11.

Supplementary Material 12.

Supplementary Material 13.

Supplementary Material 14.

Supplementary Material 15.

Supplementary Material 16.

Supplementary Material 17.

Supplementary Material 18.

Supplementary Material 19.

## Data Availability

The survey data are publicly available on the internet for data users and researchers throughout the world ( www.cdc.gov/nchs/nhanes/ ).

## Abbreviations

95% CI: 95% confidence interval
ALT: Alanine aminotransferase
AST: Aspartate aminotransferase
AUC: Area under the curve
BMI: Body mass index
BNP: B-type natriuretic peptide
CHF: Congestive heart failure
CRP: C-reactive protein
DBP: Diastolic blood pressure
eGFR: Estimated glomerular filtration rate
FPG: Fasting plasma glucose
HbA1c: Glycated hemoglobin A1c
HDL-C: High-density lipoprotein cholesterol
HFrEF: Heart failure with reduced ejection fraction
HFpEF: Heart failure with preserved ejection fraction
IL: Interleukin
IQR: Interquartile range
LASSO: Least absolute shrinkage and selection operator
LHR: Lymphocyte to high-density lipoprotein ratio
MACE: Major adverse cardiovascular events
ML: Machine learning
NCHS: National Center for Health Statistics
NHANES: National Health and Nutrition Examination Survey
NHR: Neutrophil to high-density lipoprotein cholesterol ratio
NLR: Neutrophil-to-lymphocyte ratio
NO: Nitric oxide
OGTT: Oral glucose tolerance test
OR: Odds ratio
PHR: Platelet to high-density lipoprotein ratio
PKG: Protein kinase G
PLR: Platelet to lymphocyte ratio
RAS: Renin-angiotensin system
ROC: Receiver operating characteristic curve
ROS: Reactive oxygen species
SBP: Systolic blood pressure
SII: Systemic immune-inflammation index
TC: Total cholesterol
TG: Triglycerides
TLR4: Toll-like receptor 4
TNF-α: Tumor necrosis factor-alpha
VIF: Variance inflation factor
