# Supplementary material for: Neutrophil to high-density lipoprotein cholesterol ratio is positively associated with congestive heart failure among hypertensive adults: a population-based study
Source: BMC Cardiovasc Disord. 2026 Jul 23;26:943. doi: 10.1186/s12872-026-06177-x (PMC13682066; doi:10.1186/s12872-026-06177-x)
Supplement: Supplementary file 1 [file 12872_2026_6177_MOESM10_ESM.docx]

**Table S3** Comparison of AUCs between indicators using DeLong’s test.

| **Comparisons** | **AUC 1** | **AUC 2** | **Z value** | ***P* value** |
| --- | --- | --- | --- | --- |
| **NHR and LHR** | 0.588 | 0.519 | 4.677 | <0.001 |
| **NHR and PHR** | 0.588 | 0.519 | 4.589 | <0.001 |
| **NHR and SII** | 0.588 | 0.533 | 6.083 | <0.001 |
| **NHR and PLR** | 0.588 | 0.500 | 7.159 | <0.001 |
| **NHR and NLR** | 0.588 | 0.601 | -1.398 | 0.162 |

Abbreviations: AUC, Area Under the Curve; NHR, Neutrophil to high-density lipoprotein cholesterol ratio; LHR, lymphocyte to high-density lipoprotein ratio; PHR, platelet to high-density lipoprotein ratio; PLR, platelet to lymphocyte ratio; NLR, Neutrophil to lymphocyte ratio; SII, systemic immune-inflammation index.
