# Supplementary material for: Neutrophil to high-density lipoprotein cholesterol ratio is positively associated with congestive heart failure among hypertensive adults: a population-based study
Source: BMC Cardiovasc Disord. 2026 Jul 23;26:943. doi: 10.1186/s12872-026-06177-x (PMC13682066; doi:10.1186/s12872-026-06177-x)
Supplement: Supplementary file 2 [file 12872_2026_6177_MOESM11_ESM.docx]

**Table S4** Optimal hyperparameters for machine learning models.

| **Model** | **Hyperparameter** | | | | |
| --- | --- | --- | --- | --- | --- |
| **DT** | ccp_alpha | max_depth | max_features | min_samples_split |  |
|  | 0.0 | 5 | None | 10 |  |
| **RF** | n_estimators | max_features |  |  |  |
|  | 400 | 2 |  |  |  |
| **XGBoost** | learning_rate | max_depth | n_estimators | subsample |  |
|  | 0.1 | 3 | 50 | 0.6 |  |
| **LightGBM** | colsample_bytree | learning_rate | n_estimators | num_leaves | subsample |
|  | 0.6 | 0.1 | 100 | 31 | 0.6 |
| **SVM** | C | degree | gamma | kernel |  |
|  | 0.1 | 2 | auto | rbf |  |
| **ANN** | activation | hidden_layer_sizes |  |  |  |
|  | relu | (50,) |  |  |  |

Abbreviations: DT, Decision Tree; RF, Random Forest; XGBoost, Extreme Gradient Boosting; LightGBM, Light Gradient Boosting Machine; SVM, Support Vector Machine; ANN, Artificial Neural Network.
