# Supplementary material for: Neutrophil to high-density lipoprotein cholesterol ratio is positively associated with congestive heart failure among hypertensive adults: a population-based study
Source: BMC Cardiovasc Disord. 2026 Jul 23;26:943. doi: 10.1186/s12872-026-06177-x (PMC13682066; doi:10.1186/s12872-026-06177-x)
Supplement: Supplementary file 3 [file 12872_2026_6177_MOESM12_ESM.docx]

**Table S5** Associations between NHR and CHF in the overall population.

| **NHR** | **CHF** | | |
| --- | --- | --- | --- |
|  | **OR** | **95% CI** | ***P*** |
| **Crude model (Model 1)** |  | | |
| **Continuous** | 1.26 | 1.21, 1.31 | <0.001 |
| **Categories** |  | | |
| Quartile 1 | 1 (ref) | | |
| Quartile 2 | 1.29 | 1.04, 1.61 | 0.022 |
| Quartile 3 | 1.64 | 1.31, 2.05 | <0.001 |
| Quartile 4 | 2.44 | 1.98, 2.99 | <0.001 |
| *P* for tend | 1.92 | 1.65, 2.22 | <0.001 |
| **Minimally adjusted model (Model 2)** |  | | |
| **Continuous** | 1.34 | 1.28, 1.40 | <0.001 |
| **Categories** |  | | |
| Quartile 1 | 1 (ref) | | |
| Quartile 2 | 1.42 | 1.14, 1.78 | 0.002 |
| Quartile 3 | 1.95 | 1.54, 2.47 | <0.001 |
| Quartile 4 | 3.14 | 2.51, 3.92 | <0.001 |
| *P* for tend | 2.31 | 1.97, 2.71 | <0.001 |
| **Fully adjusted model (Model 3)** |  | | |
| **Continuous** | 1.12 | 1.06, 1.17 | <0.001 |
| **Categories** |  | | |
| Quartile 1 | 1 (ref) | | |
| Quartile 2 | 1.02 | 0.81, 1.29 | 0.900 |
| Quartile 3 | 1.15 | 0.90, 1.48 | 0.300 |
| Quartile 4 | 1.39 | 1.10, 1.76 | 0.006 |
| *P* for tend | 1.28 | 1.08, 1.53 | 0.005 |

The neutrophil to high-density lipoprotein cholesterol ratio was converted from a continuous variable to a categorical variable (quartiles). All regression coefficients, ORs, and 95% CIs are weighted estimates based on survey-weighted logistic regression. Abbreviations: OR, odds ratio; 95% CI, 95% confidence interval; NHR, neutrophil to high-density lipoprotein cholesterol ratio; CHF, Congestive Heart Failure. Model 1: no covariates were adjusted. Model 2: age, sex, and race were adjusted. Model 3: age, sex, race, education level, marital status, family PIR, BMI, [smoked at least 100 cigarettes in life](https://wwwn.cdc.gov/Nchs/Data/Nhanes/Public/2013/DataFiles/SMQ_H.htm#SMQ020), drinking frequency, diabetes, the use of glucose-lowering medications, lipid-lowering medications, antihypertensive medications, and heart failure medications, FPG, HbA_1c_, ALB, ALT, AST, serum creatinine, and eGFR were adjusted.
