# Supplementary material for: Neutrophil to high-density lipoprotein cholesterol ratio is positively associated with congestive heart failure among hypertensive adults: a population-based study
Source: BMC Cardiovasc Disord. 2026 Jul 23;26:943. doi: 10.1186/s12872-026-06177-x (PMC13682066; doi:10.1186/s12872-026-06177-x)
Supplement: Supplementary file 8 [file 12872_2026_6177_MOESM17_ESM.docx]

**Table S10** Associations between NHR and CHF.

| **NHR** | **CHF** | | |
| --- | --- | --- | --- |
|  | **OR** | **95% CI** | ***P*** |
| **Crude model (Model 1)** |  | | |
| **Continuous** | 1.21 | 1.16, 1.27 | <0.001 |
| **Categories** |  | | |
| Quartile 1 | 1 (ref) | | |
| Quartile 2 | 1.30 | 1.05, 1.61 | 0.015 |
| Quartile 3 | 1.72 | 1.41, 2.09 | <0.001 |
| Quartile 4 | 2.26 | 1.84, 2.78 | <0.001 |
| *P* for tend | 1.84 | 1.59, 2.12 | <0.001 |
| **Minimally adjusted model (Model 2)** |  | | |
| **Continuous** | 1.31 | 1.25, 1.37 | <0.001 |
| **Categories** |  | | |
| Quartile 1 | 1 (ref) | | |
| Quartile 2 | 1.44 | 1.15, 1.79 | 0.001 |
| Quartile 3 | 2.05 | 1.67, 2.53 | <0.001 |
| Quartile 4 | 3.02 | 2.42, 3.77 | <0.001 |
| *P* for tend | 2.27 | 1.95, 2.65 | <0.001 |
| **Fully adjusted model (Model 3)** |  | | |
| **Continuous** | 1.14 | 1.09, 1.20 | <0.001 |
| **Categories** |  | | |
| Quartile 1 | 1 (ref) | | |
| Quartile 2 | 1.10 | 0.88, 1.39 | 0.400 |
| Quartile 3 | 1.41 | 1.13, 1.77 | 0.003 |
| Quartile 4 | 1.65 | 1.31, 2.08 | <0.001 |
| *P* for tend | 1.48 | 1.26, 1.74 | <0.001 |

The neutrophil to high-density lipoprotein cholesterol ratio was converted from a continuous variable to a categorical variable (quartiles). All regression coefficients, ORs, and 95% CIs are weighted estimates based on survey-weighted logistic regression. Abbreviations: OR, odds ratio; 95% CI, 95% confidence interval; NHR, neutrophil to high-density lipoprotein cholesterol ratio; CHF, Congestive Heart Failure. Model 1: no covariates were adjusted. Model 2: age, sex, and race were adjusted. Model 3: age, sex, race, education level, marital status, family PIR, BMI, [smoked at least 100 cigarettes in life](https://wwwn.cdc.gov/Nchs/Data/Nhanes/Public/2013/DataFiles/SMQ_H.htm#SMQ020), drinking frequency, diabetes, the use of glucose-lowering medications, lipid-lowering medications, antihypertensive medications, and heart failure medications, FPG, HbA_1c_, ALB, ALT, AST, serum creatinine, and eGFR were adjusted. PIR: < 1, very low income; 1-1.99, low income; 2-3.99, middle income; ≥ 4, high income. BMI: 18.5-24.9, normal; < 18.5 or ≥ 25, abnormal.
