# Supplementary material for: Neutrophil to high-density lipoprotein cholesterol ratio is positively associated with congestive heart failure among hypertensive adults: a population-based study
Source: BMC Cardiovasc Disord. 2026 Jul 23;26:943. doi: 10.1186/s12872-026-06177-x (PMC13682066; doi:10.1186/s12872-026-06177-x)
Supplement: Supplementary file 9 [file 12872_2026_6177_MOESM18_ESM.docx]

**Table S11** Associations between NHR and CHF based on unweighted analyses.

| **NHR** | **CHF** | | |
| --- | --- | --- | --- |
|  | **OR** | **95% CI** | ***P*** |
| **Crude model (Model 1)** |  | | |
| **Continuous** | 1.21 | 1.17, 1.25 | <0.001 |
| **Categories** |  | | |
| Quartile 1 | 1 (ref) | | |
| Quartile 2 | 1.34 | 1.11, 1.61 | 0.002 |
| Quartile 3 | 1.57 | 1.31, 1.88 | <0.001 |
| Quartile 4 | 2.26 | 1.91, 2.68 | <0.001 |
| *P* for tend | 1.79 | 1.59, 2.02 | <0.001 |
| **Minimally adjusted model (Model 2)** |  | | |
| **Continuous** | 1.27 | 1.23, 1.32 | <0.001 |
| **Categories** |  | | |
| Quartile 1 | 1 (ref) | | |
| Quartile 2 | 1.45 | 1.20, 1.75 | <0.001 |
| Quartile 3 | 1.79 | 1.49, 2.16 | <0.001 |
| Quartile 4 | 2.75 | 2.30, 3.30 | <0.001 |
| *P* for tend | 2.07 | 1.82, 2.35 | <0.001 |
| **Fully adjusted model (Model 3)** |  | | |
| **Continuous** | 1.13 | 1.08, 1.17 | <0.001 |
| **Categories** |  | | |
| Quartile 1 | 1 (ref) | | |
| Quartile 2 | 1.17 | 0.97, 1.43 | 0.110 |
| Quartile 3 | 1.29 | 1.06, 1.57 | 0.012 |
| Quartile 4 | 1.61 | 1.33, 1.96 | <0.001 |
| *P* for tend | 1.40 | 1.22, 1.62 | <0.001 |

The neutrophil to high-density lipoprotein cholesterol ratio was converted from a continuous variable to a categorical variable (quartiles). Abbreviations: OR, odds ratio; 95% CI, 95% confidence interval; NHR, neutrophil to high-density lipoprotein cholesterol ratio; CHF, Congestive Heart Failure. Model 1: no covariates were adjusted. Model 2: age, sex, and race were adjusted. Model 3: age, sex, race, education level, marital status, family PIR, BMI, [smoked at least 100 cigarettes in life](https://wwwn.cdc.gov/Nchs/Data/Nhanes/Public/2013/DataFiles/SMQ_H.htm#SMQ020), drinking frequency, diabetes, the use of glucose-lowering medications, lipid-lowering medications, antihypertensive medications, and heart failure medications, FPG, HbA_1c_, ALB, ALT, AST, serum creatinine, and eGFR were adjusted.
