# Supplementary material for: Neutrophil to high-density lipoprotein cholesterol ratio is positively associated with congestive heart failure among hypertensive adults: a population-based study
Source: BMC Cardiovasc Disord. 2026 Jul 23;26:943. doi: 10.1186/s12872-026-06177-x (PMC13682066; doi:10.1186/s12872-026-06177-x)
Supplement: Supplementary file 10 [file 12872_2026_6177_MOESM19_ESM.docx]

**Table S12** Associations between NLR and CHF.

| **NHR** | **CHF** | | |
| --- | --- | --- | --- |
|  | **OR** | **95% CI** | ***P*** |
| **Crude model (Model 1)** |  | | |
| **Continuous** | 1.26 | 1.21, 1.32 | <0.001 |
| **Categories** |  | | |
| Quartile 1 | 1 (ref) | | |
| Quartile 2 | 0.98 | 0.78, 1.22 | 0.800 |
| Quartile 3 | 1.23 | 1.00, 1.51 | 0.055 |
| Quartile 4 | 2.28 | 1.90, 2.73 | <0.001 |
| *P* for tend | 1.83 | 1.60, 2.09 | <0.001 |
| **Minimally adjusted model (Model 2)** |  | | |
| **Continuous** | 1.22 | 1.17, 1.28 | <0.001 |
| **Categories** |  | | |
| Quartile 1 | 1 (ref) | | |
| Quartile 2 | 1.00 | 0.80, 1.25 | >0.900 |
| Quartile 3 | 1.25 | 1.01, 1.54 | 0.040 |
| Quartile 4 | 2.07 | 1.71, 2.50 | <0.001 |
| *P* for tend | 1.71 | 1.49, 1.96 | <0.001 |
| **Fully adjusted model (Model 3)** |  | | |
| **Continuous** | 1.13 | 1.08, 1.18 | <0.001 |
| **Categories** |  | | |
| Quartile 1 | 1 (ref) | | |
| Quartile 2 | 0.92 | 0.74, 1.15 | 0.500 |
| Quartile 3 | 1.05 | 0.84, 1.31 | 0.700 |
| Quartile 4 | 1.49 | 1.22, 1.82 | <0.001 |
| *P* for tend | 1.34 | 1.16, 1.56 | <0.001 |

The neutrophil to high-density lipoprotein cholesterol ratio was converted from a continuous variable to a categorical variable (quartiles). All regression coefficients, ORs, and 95% CIs are weighted estimates based on survey-weighted logistic regression. Abbreviations: OR, odds ratio; 95% CI, 95% confidence interval; NHR, neutrophil to high-density lipoprotein cholesterol ratio; CHF, Congestive Heart Failure. Model 1: no covariates were adjusted. Model 2: age, sex, and race were adjusted. Model 3: age, sex, race, education level, marital status, family PIR, BMI, [smoked at least 100 cigarettes in life](https://wwwn.cdc.gov/Nchs/Data/Nhanes/Public/2013/DataFiles/SMQ_H.htm#SMQ020), drinking frequency, diabetes, the use of glucose-lowering medications, lipid-lowering medications, antihypertensive medications, and heart failure medications, FPG, HbA_1c_, ALB, ALT, AST, serum creatinine, and eGFR were adjusted.
