# Supplementary material for: Neutrophil to high-density lipoprotein cholesterol ratio is positively associated with congestive heart failure among hypertensive adults: a population-based study
Source: BMC Cardiovasc Disord. 2026 Jul 23;26:943. doi: 10.1186/s12872-026-06177-x (PMC13682066; doi:10.1186/s12872-026-06177-x)
Supplement: Supplementary file 11 [file 12872_2026_6177_MOESM8_ESM.docx]

**Table S1** Baseline characteristics of the study population according to CHF.

| **Characteristics** | **Overall**  N = 72,910,237 | **Without CHF**  N = 69,304,535 | **With CHF**  N = 3,605,702 | ***p*-value** |
| --- | --- | --- | --- | --- |
| **Male,** (%) | 37,815,420 (52%) | 35,994,080 (52%) | 1,821,341 (51%) | 0.414 |
| **Age** ≥ 60**,** (%) | 38,649,104 (53%) | 37,768,646 (54%) | 880,457 (24%) | <0.001 |
| **Race,** (%) |  |  |  | 0.012 |
| Mexican American | 4,076,731 (5.6%) | 3,940,163 (5.7%) | 136,568 (3.8%) |  |
| Other Hispanic | 3,245,109 (4.5%) | 3,092,251 (4.5%) | 152,857 (4.2%) |  |
| Non-Hispanic White | 51,531,971 (71%) | 48,964,628 (71%) | 2,567,344 (71%) |  |
| Non-Hispanic Black | 9,627,638 (13%) | 9,044,171 (13%) | 583,467 (16%) |  |
| Other Races | 4,428,788 (6.1%) | 4,263,323 (6.2%) | 165,466 (4.6%) |  |
| **Education level,** (%) |  |  |  | <0.001 |
| < high school | 5,473,041 (7.5%) | 5,001,369 (7.2%) | 471,673 (13%) |  |
| High school | 8,971,687 (12%) | 8,282,639 (12%) | 689,048 (19%) |  |
| > high school | 58,465,509 (80%) | 56,020,528 (81%) | 2,444,982 (68%) |  |
| **[Marital status](https://wwwn.cdc.gov/Nchs/Data/Nhanes/Public/2005/DataFiles/DEMO_D.htm#DMDMARTL),** (%) |  |  |  | <0.001 |
| Married | 46,378,747 (64%) | 44,435,616 (64%) | 1,943,132 (54%) |  |
| Widowed | 8,786,781 (12%) | 7,884,954 (11%) | 901,827 (25%) |  |
| Divorced | 10,629,956 (15%) | 10,064,399 (15%) | 565,557 (16%) |  |
| Never married | 7,114,754 (9.8%) | 6,919,567 (10.0%) | 195,187 (5.4%) |  |
| **Family PIR,** (%) |  |  |  | <0.001 |
| Low income(<1) | 9,726,120 (13%) | 8,993,523 (13%) | 732,597 (20%) |  |
| Median income(1-3.9) | 37,139,981 (51%) | 34,865,557 (50%) | 2,274,424 (63%) |  |
| High income(≥4) | 26,044,136 (36%) | 25,445,455 (37%) | 598,681 (17%) |  |
| **BMI,** (%) |  |  |  | <0.001 |
| <18.5 | 34,314,961 (47%) | 32,354,224 (47%) | 1,960,738 (54%) |  |
| 18.5-24.9 | 24,326,896 (33%) | 23,299,621 (34%) | 1,027,275 (28%) |  |
| 25-29.9 | 13,490,651 (19%) | 12,904,750 (19%) | 585,900 (16%) |  |
| ≥30 | 777,729 (1.1%) | 745,940 (1.1%) | 31,789 (0.9%) |  |
| **Diabetes,** (%) | 16,983,220 (23%) | 15,266,046 (22%) | 1,717,174 (48%) | <0.001 |
| **[Smoked at least 100 cigarettes in life](https://wwwn.cdc.gov/Nchs/Data/Nhanes/Public/2013/DataFiles/SMQ_H.htm#SMQ020),** (%) | 36,234,894 (50%) | 34,090,789 (49%) | 2,144,104 (59%) | <0.001 |
| **Drinking frequency,** (%) |  |  |  | <0.001 |
| Weekly | 30,714,470 (42%) | 29,433,317 (42%) | 1,281,153 (36%) |  |
| Monthly | 16,280,951 (22%) | 15,582,536 (22%) | 698,415 (19%) |  |
| Yearly | 25,914,816 (36%) | 24,288,682 (35%) | 1,626,134 (45%) |  |
| **The use of antihypertensive medications,** (%) | 50,125,823 (69%) | 46,864,049 (68%) | 3,261,774 (90%) | <0.001 |
| **The use of lipid-lowering medications,** (%) | 26,469,538 (36%) | 24,350,486 (35%) | 2,119,052 (59%) | <0.001 |
| **The use of glucose-lowering medications,** (%) | 11,034,005 (15%) | 9,711,554 (14%) | 1,322,451 (37%) | <0.001 |
| **The use of heart failure medications,** (%) | 35,415,243 (49%) | 32,491,109 (47%) | 2,924,134 (81%) | <0.001 |
| **Albumin** (g/dL) | 4.22± (0.34) | 4.23± (0.34) | 4.06± (0.36) | <0.001 |
| **ALT** (U/L) | 26.29± (20.03) | 26.39± (17.89) | 24.25± (44.29) | <0.001 |
| **AST** (U/L) | 26.21± (16.38) | 26.22± (15.97) | 26.03± (22.84) | 0.070 |
| **Serum creatinine** (mg/dL) | 0.95± (0.53) | 0.93± (0.49) | 1.24± (0.98) | <0.001 |
| **eGFR** (ml/min/1.73m²) | 83.01± (22.34) | 83.95± (21.76) | 64.98± (25.58) | <0.001 |
| **FPG** (mmol/L) | 6.30± (2.12) | 6.27± (2.09) | 6.83± (2.48) | <0.001 |
| **HbA_1c_** (%) | 5.84± (1.07) | 5.82± (1.06) | 6.28± (1.28) | <0.001 |
| **HDL-C** (mmol/L) | 1.37± (0.43) | 1.38± (0.43) | 1.28± (0.40) | <0.001 |
| **TG** (mmol/L) | 1.69± (1.22) | 1.69± (1.23) | 1.74± (1.18) | 0.147 |
| **TC** (mmol/L) | 5.16± (1.11) | 5.18± (1.10) | 4.73± (1.19) | <0.001 |
| **NHR** | 3.43± (1.54) | 3.41± (1.53) | 3.91± (1.60) | <0.001 |
| **LHR** | 1.70± (1.36) | 1.70± (1.34) | 1.73± (1.66) | 0.181 |
| **PHR** | 197.98± (76.49) | 198.27± (76.47) | 192.38± (76.79) | 0.054 |
| **PLR** | 132.42± (55.65) | 132.32± (54.66) | 134.36± (72.01) | 0.187 |
| **NLR** | 2.29± (1.19) | 2.26± (1.15) | 2.77± (1.76) | <0.001 |
| **SII** | 573.00± (348.05) | 569.77± (337.44) | 635.09± (506.97) | 0.005 |

All continuous variables were presented as survey-weighted means and standard deviations, while categorical variables were expressed as survey-weighted frequencies and percentages. P values were calculated using survey-weighted chi-square tests for categorical variables and survey-weighted linear regression for continuous variables. Abbreviations: NHR, Neutrophil to high-density lipoprotein cholesterol ratio; Q, quartiles; PIR, Poverty Income Ratio; BMI, Body Mass Index; HDL-C, high-density lipoprotein cholesterol; TG, triglycerides; TC, total cholesterol; FPG, fasting plasma glucose; HbA_1c_, glycated hemoglobin A1c; ALT, alanine aminotransferase; AST, aspartate aminotransferase; eGFR, estimated glomerular filtration rate; LHR, lymphocyte to high-density lipoprotein ratio; PHR, platelet to high-density lipoprotein ratio; PLR, platelet to lymphocyte ratio; NLR, Neutrophil to lymphocyte ratio; SII, systemic immune-inflammation index.
