# Supplementary material for: Neutrophil to high-density lipoprotein cholesterol ratio is positively associated with congestive heart failure among hypertensive adults: a population-based study
Source: BMC Cardiovasc Disord. 2026 Jul 23;26:943. doi: 10.1186/s12872-026-06177-x (PMC13682066; doi:10.1186/s12872-026-06177-x)
Supplement: Supplementary file 12 [file 12872_2026_6177_MOESM9_ESM.docx]

**Table S2** Variance Inflation Factors (VIFs) of covariates in the regression model.

| **Variables** | **VIF** |
| --- | --- |
| HbA1c | 3.651 |
| FPG | 3.157 |
| Race (Non-Hispanic White) | 2.872 |
| Diabetes | 2.850 |
| Race (Non-Hispanic Black) | 2.633 |
| ALT | 2.626 |
| eGFR | 2.612 |
| The use of glucose-lowering medications | 2.556 |
| AST | 2.461 |
| Education level (> high school) | 2.309 |
| Family PIR [High income (≥4)] | 2.097 |
| Education level (high school) | 1.922 |
| Family PIR [Median income (1-3.9)] | 1.863 |
| Serum creatinine | 1.827 |
| Age (< 60 years) | 1.754 |
| Race (Other Races) | 1.548 |
| The use of heart failure medications | 1.541 |
| The use of antihypertensive medications | 1.497 |
| Drinking frequency (Yearly) | 1.450 |
| Race (Other Hispanic) | 1.438 |
| Sex (Male) | 1.375 |
| BMI (25-29.9) | 1.341 |
| Drinking frequency (Monthly) | 1.308 |
| Marital status (Widowed) | 1.293 |
| BMI (18.5-24.9) | 1.277 |
| NHR | 1.247 |
| The use of lipid-lowering medications | 1.209 |
| Albumin | 1.176 |
| Marital status (Never married) | 1.155 |
| Marital status (Divorced) | 1.144 |
| Smoked at least 100 cigarettes in life | 1.113 |
| BMI (≥30) | 1.040 |

Abbreviations: FPG, fasting plasma glucose; HbA_1c_, glycated hemoglobin A1c; ALT, alanine aminotransferase; AST, aspartate aminotransferase; eGFR, estimated glomerular filtration rate; PIR, Poverty Income Ratio; BMI, Body Mass Index; NHR, Neutrophil to high-density lipoprotein cholesterol ratio; VIF, Variance Inflation Factor.
